# Swine Influenza A Vaccines, Pandemic (H1N1) 2009 Virus, and Cross-Reactivity

**DOI:** 10.3201/eid1606.100138

**Published:** 2010-06

**Authors:** Ralf Dürrwald, Andi Krumbholz, Sigrid Baumgarte, Michael Schlegel, Thomas W. Vahlenkamp, Hans-Joachim Selbitz, Peter Wutzler, Roland Zell

**Affiliations:** IDT Biologika GmbH, Dessau Rosslau, Germany (R. Dürrwald, M. Schlegel, H.-J. Selbitz); Universitätsklinikum Jena, Jena, Germany (A. Krumbholz, P. Wutzler, R. Zell); Institut für Umwelt und Hygiene, Hamburg, Germany (S. Baumgarte); Friedrich-Loeffler-Institut, Insel Riems, Germany (T.W. Vahlenkamp)

**Keywords:** Viruses, influenza, pandemic (H1N1) 2009, swine, vaccines, pandemic, cross-reactivity, expedited, letter

**To the Editor:** Since its first emergence in the human population in spring 2009 (*1*–*3*) infections with pandemic (H1N1) 2009 virus have been reported in pigs, turkeys, and some carnivore species (*4*,*5*). The pandemic (H1N1) 2009 virus can be experimentally transmitted between pigs (*6*). The reported transmissibility of the virus raises the question as to whether authorized swine influenza vaccine strains may be cross-reactive to pandemic (H1N1) 2009 virus. Kyriakis et al. (*7*) investigated the cross-reactivity of 66 pig serum samples from different infection and vaccination trials and reported cross-reactions between the avian-like H1N1 viruses circulating in the European pig population (avH1N1) and the classical swine H1N1 viruses (cH1N1) with pandemic (H1N1) 2009 virus by hemagglutination inhibition assay.

To investigate this cross-reactivity in more detail, a neutralization test was applied in the study we report here. A serial dilution of serum samples was prepared (log_4_). All virus strains were adjusted to 100 fifty-percent tissue culture infectious doses. This working dilution of virus was mixed with serum dilutions and incubated 1 hour at 37°C. Madin-Darby bovine kidney monolayers were infected with the neutralization mixtures. After 48 hours of incubation, cells were fixed with acetone (4°C–8°C) and investigated by indirect immunofluorescent assay. Finally, the 50% neutralization titer was calculated.

Hyperimmune serum samples were established by using a 4-fold vaccination of pigs with antigens of H1N1 vaccine strains (A/New Jersey/8/1976, A/sw/Netherlands/25/1980, A/sw/IDT/Re230/1992, A/sw/Haselünne/IDT2617/2003), and a strain of pandemic (H1N1) 2009 virus (A/Hamburg/7/2009) by using Freund adjuvant. Blood samples were taken 14 days after last immunization. A vaccine containing the pandemic (H1N1) 2009 virus was produced. Swine influenza vaccines available in central Europe and the newly produced vaccine containing pandemic (H1N1) 2009 virus (A/Hamburg/7/2009) were administered to pigs (2-fold vaccination with 1–2 mL of the vaccine 21–28 days apart intramuscularly). Blood was withdrawn 7 days after second administration.

In addition, an experimental aerosol infection was conducted by using the parental strain of the most recent avH1N1 strain contained in a European swine influenza vaccine (A/sw/Haselünne/IDT2617/2003). Blood samples were taken 10 days after infection.

The investigation of the hyperimmune serum samples detected neutralizing activity between the pandemic (H1N1) 2009 virus and European avH1N1 vaccine strains (A/sw/Netherlands/25/1980, A/sw/IDT/Re230/1992, A/sw/Haselünne/IDT2617/2003), as well as with the cH1N1 strain A/New Jersey/8/1976 (Fort Dix reassortant). The hyperimmune serum established against pandemic (H1N1) 2009 virus also showed cross-reactivity with European avH1N1 virus. The reactions against several strains of the pandemic virus were similar, reflecting high titers against pandemic (H1N1) 2009 virus but also cross-reactions with hyperimmune serum samples of all swine influenza A virus H1N1 vaccine strains (Appendix Table).

The bivalent vaccines induced high titers of neutralizing antibodies against avH1N1 virus and human-like H3N2 virus (huH3N2). Only a low number of pigs reacted with H1N2 virus whereas the trivalent vaccine induced high neutralizing activity in serum samples of all vaccinated pigs. The vaccines induced neutralizing antibodies against pandemic (H1N1) 2009 virus. The titers were lower in comparison to those obtained for avH1N1 and not all pigs responded. The reactions were best for the vaccines containing mineral oil. Pigs vaccinated with the trivalent vaccine with carbomer adjuvant showed almost no antibodies against pandemic (H1N1) 2009 virus, although the vaccine strain reacted well in hyperimmunization tests.

A vaccine batch of the trivalent vaccine was produced that contained mineral oil instead of carbomer. All pigs vaccinated with the trivalent vaccine with mineral oil had antibodies against the pandemic (H1N1) 2009 virus (data not shown). At the same time, efficacy trials with all authorized vaccines were conducted (*8*; T.W. Vahlenkamp, pers. comm.) in which all vaccines including the trivalent vaccine with carbomer adjuvant showed a comparable level of protection (limited period of viral shedding). Mineral oil adjuvants can induce severe distress in pig herds due to their limited safety. Despite cross-reactivity between avH1N1 and cH1N1 with pandemic (H1N1) 2009 virus, the highest degree of cross-neutralization was achieved by the vaccine containing pandemic (H1N1) virus strain.

Proof of cross-reactivity was also reflected in the infection trial. Pigs infected with avH1N1 responded to avH1N1 as well as to pandemic (H1N1) 2009 virus. All results were additionally confirmed by hemagglutination inhibition assay (data not shown).

Furthermore, 1,559 pig serum samples from 195 German pig herds collected from mid-June through mid-September 2009 were tested in routine diagnostics by hemagglutination inhibition assay. All reflected almost similar results for avH1N1 and the pandemic (H1N1) 2009 virus (seroprevalences for individual pigs were pandemic [H1N1] 2009 virus 52%, avH1N1 53%, huH1N2 28%, and huH3N2 52%; for pig herds pandemic [H1N1] 2009 virus 46%, avH1N1 46%, huH1N2 24%, and huH3N2 45%). These results suggest cross-reactivity between porcine H1N1 viruses and pandemic (H1N1) 2009 virus. Despite this cross-reactivity, a vaccine consisting of pandemic (H1N1) 2009 virus is superior in terms of efficacy in comparison with vaccines already authorized in Europe.

## Supplementary Material

Appendix Table Serologic cross-reactivity between vaccine strains used in European swine influenza A virus vaccines and pandemic (H1N1) 2009 virus and influenza A viruses currently circulating in the human population, measured by neutralization test*

## References

[1] Garten RJ, Davis CT, Russell CA, Shu B, Lindstrom S, Balish A, Antigenic and genetic characteristics of swine-origin 2009 A(H1N1) influenza viruses circulating in humans. Science. 2009;325:197–201. 10.1126/science.1176225 19465683 PMC3250984

[2] Smith GJ, Vijaykrishna D, Bahl J, Lycett SJ, Worobey M, Pybus OG, Origins and evolutionary genomics of the 2009 swine-origin H1N1 influenza A epidemic. Nature. 2009;459:1122–5. 10.1038/nature08182 19516283

[3] Itoh Y, Shinya K, Kiso M, Watanabe T, Sakoda Y, Hatta M, In vitro and in vivo characterization of new swine-origin H1N1 influenza viruses. Nature. 2009;460:1021–5. 19672242 10.1038/nature08260 PMC2748827

[4] Influenza pandemic (H1N1), animal. Latest information on influenza A (H1N1) [cited 2010 Jan 31]. http://www.promedmail.org

[5] Hofshagen M, Gjerset B, Er C, Tarpai A, Brun E, Dannevig B, Pandemic influenza A(H1N1)v: Human to pig transmission in Norway? Euro Surveill. 2009; 14:pii=19406. 10.2807/ese.14.45.19406-en 19941789

[6] Brookes SM, Irvine RM, Nunez A, Clifford D, Essen S, Brown IH, Influenza A (H1N1) infection in pigs. Vet Rec. 2009;164:760–1. 10.1136/vr.164.24.760 19525527

[7] Kyriakis CS, Olsen CW, Carman S, Brown IH, Brookes SM, Van Doorsselaere J, Serologic cross-reactivity with pandemic (H1N1) 2009 virus in pigs, Europe. Emerg Infect Dis. 2010;16:96–9. 10.3201/eid1601.091190 20031049 PMC2874381

[8] Lange E, Kalthoff D, Blohm U, Teifke JP, Breithaupt A, Maresch C, Pathogenesis and transmission of the novel swine-origin influenza virus A/H1N1 after experimental infection of pigs. J Gen Virol. 2009;90:2119–23. 10.1099/vir.0.014480-0 19592456

