# Supplementary material for: Swine Influenza A Vaccines, Pandemic (H1N1) 2009 Virus, and Cross-Reactivity
Source: Emerg Infect Dis. 2010 Jun;16(6):1029–30. doi: 10.3201/eid1606.100138 (PMC3086252; doi:10.3201/eid1606.100138)
Supplement: Appendix Table — Serologic cross-reactivity between vaccine strains used in European swine influenza A virus vaccines and pandemic (H1N1) 2009 virus and influenza A viruses currently circulating in the human population, measured by neutralization test* [file 10-0138-appT-s1.pdf]

Publisher: CDC; Journal: Emerging Infectious Diseases
Article Type: Letter; Volume: 16; Issue: 6; Year: 2010; Article ID: 10-0138
DOI: 10.3201/eid1606.100138; TOC Head: Letter

Appendix Table. Serologic cross-reactivity between vaccine strains used in European swine influenza A virus vaccines and pandemic (H1N1) 2009 virus and influenza A viruses currently circulating in the human population, measured by neutralization test*

		Range of antibody titers (no. pigs with neutralization test antibodies >13)							
		European swine influenza A virus			Pandemic (H1N1) 2009 virus			Human influenza A virus	
Group	No. pigs	Swine/Haselünne/ IDT2617/2003 (avH1N1)	Swine/Bakum/ 1832/2000 (huH1N2)	Swine/Bakum/ IDT1769/2003 (huH3N2)	Hamburg/7/2009 (H1N1)	Jena/5258/2009 (H1N1)	Jena/5555/2009 (H1N1)	Brisbane/59/2007 (H1N1)	Brisbane/10/2007 (H3N2)
Hyperimmunization†									
New Jersey/8/76 (cH1N1)	1	1,288 (1)	324 (1)	–‡	3,236 (1)	1,288 (1)	1,288 (1)	–	–
Swine/Netherlands/25/80 (avH1N1)	1	1,288 (1)	–	–	129 (1)	51 (1)	513 (1)	–	–
Swine/IDT/Re230/92 (avH1N1)	1	1,288 (1)	–	–	813 (1)	513 (1)	1,288 (1)	–	–
Swine/Haselünne/IDT2617/2003 (avH1N1)	1	8,128 (1)	–	–	3,236 (1)	1,288 (1)	5,129 (1)	–	–
Hamburg/7/2009§ (pandemic [H1N1] 2009)	2	513–813 (2)	129–324 (2)	–	20,893–52,481 (2)	5,129–8,128 (2)	20,893 (2)	–	–
Vaccination¶									
Bivalent vaccine 1 (cH1N1+H3N2)	10	129–8,128 (10)	20–204 (8)	813–5,129 (10)	129–1,288 (10)	32–204 (9)	129–1,288 (9)	204 (1)	20 (1)
Bivalent vaccine 2 (avH1N1+H3N2)	12	81–8,128 (12)	20–129 (4)	81–2,042 (11)	204–1,288 (10)	81–513 (10)	32–513 (11)	–	–
Bivalent vaccine 3 (avH1N1+huH3N2)	20	32–2,042 (20)	32–204 (5)	81–3,236 (20)	51–129 (12)	20–204 (12)	51–204 (12)	–	–
Trivalent vaccine (avH1N1+huH1N2+huH3N2)	28	129–3,236 (28)	513–8,128 (28)	513–3,236 (28)	81–129 (3)	129 (1)	51 (3)	–	–
Monovalent pandemic (H1N1) 2009 vaccine	6	129 (1)	–	–	813–8,128(6)	513–51,29 (6)	324–3,236 (6)	–	–
Infection									
Swine/Haselünne/IDT2617/2003#	5	81–513 (5)	–	–	20–513 (5)	32–204 (4)	32–513 (4)	–	–

*A total of 47 negative controls were included in the study (2 in the hyperimmunization trials, 45 in the vaccination trials); all 47 serum samples did not react in the neutralization test (data not shown). Blood samples investigated belong to 3 different groups: 1) hyperimmune serum samples, which reflect the highest degree of cross-reaction achievable in terms of antibodies; 2) vaccination serum samples, which show the capacity of the corresponding vaccine to induce antibodies; and 3) postinfection serum samples, which demonstrate the antibody response to infection. Antibody titers follow kinetics representing a peak at a certain time after antigen contact and thereafter decrease continuously to a low level. The time point of taking blood samples was chosen to correspond to the peak, which is between days 7 and 14 after hyperimmunization (depending on the immunization scheme), between days 7–10 after second administration of inactivated vaccines, and between days 10 and 14 after infection (depending on the strain).

†The strains were named c, classical swine; hu, human-like; av, avian-like; or pandemic (H1N1) 2009, influenza A pandemic (H1N1) 2009 virus, according to the origin of the hemagglutinin in the porcine viruses.

‡Negative, 50% neutralization titer ≤13.

§The name of this strain was changed several times; the designations Hamburg/4/2009, Hamburg/5/2009, and Hamburg/7/2009 refer to the same strain.

¶Inactivated vaccines: bivalent vaccine 1 (Gripovac) contains antigens of strains A/NewJersey/8/1976 (cH1N1) and A/Port Chalmers/1/1973 (H3N2), with mineral oil used as adjuvant (batch Viraflu L 49578); bivalent vaccine 2 (Suvaxyn Flu) contains strains A/swine/Netherlands/25/80 (avH1N1) and A/Port Chalmers/1/1973 (H3N2), with $Al(OH)_3$ + mineral oil used as adjuvants (batch SK-01700); bivalent vaccine 3 (RESPIPORC FLU, identical to Ingelvac FLU) consists of strains IDT/Re230/1992 and IDT/Re220/1992, which are reassortant strains based on Belgian swine influenza viruses, with $Al(OH)_3$ + mineral oil used as adjuvants (batches 019–022 08 04); trivalent vaccine (RESPIPORC FLU3, identical with Gripovac 3) contains strains A/swine/Haselünne/IDT2617/2003 (avH1N1), A/swine/Bakum/1832/2000 (huH1N2), and A/swine/Bakum/IDT1769/2003 (huH3N2), with a carbomer adjuvant (batch 005 08 06); the monovalent pandemic vaccine consists of the pandemic (H1N1) 2009 virus strain A/Hamburg/7/2009 with a carbomer adjuvant (batch 001 07 09).

#The parental strain of the vaccine strain was used for experimental aerosol infection.
